# An analysis of the bacterial and fungal diversity of the fecal material of gopher tortoises

**DOI:** 10.17912/micropub.biology.002029

**Published:** 2026-04-14

**Authors:** Lauren M.K. Vankoningsveld, Richard W. McLaughlin, Durward L. Bevis

**Affiliations:** 1 School of Liberal Arts and Sciences, Gateway Technical College, Kenosha, Wisconsin, United States

## Abstract

The gopher tortoise ( *Gopherus polyphemus* ) is listed in the State of Florida as a threatened species. It is well known that the microbiota of the host is imperative to health promotion and disease mitigation. The diversity of the gut microbiota of the tortoise has not been extensively surveyed. &nbsp;In this study, we examined both the bacterial and fungal diversity in the fecal material of this animal using bacterial tag-encoded flexible-Titanium (FLX) amplicon pyrosequencing (bTEFAP) and fungal tag-encoded FLX amplicon pyrosequencing.&nbsp; In the six samples, there were 16 total bacterial phyla identified with Bacillota (54.55 to 86.13%) as the most dominant and two fungal phyla identified with Ascomycota (79.64 to 97.32%) as the most predominant.&nbsp; Interestingly, the pathogenic fungus *Candida tropicalis* was detected in all samples suggesting the tortoise could be a reservoir of zoonotic fungi.

**Table 1. Bacterial and fungal diversity present in the fecal material of two gopher tortoises:** An analysis of the Predicted Bacterial Phyla (1A), Predicted Bacterial Genera (1B), Bacterial Information (1C), Predicted Fungal Phyla (1D), Predicted Fungal Genera (1E), and Fungal Information (1F). The values listed in sections 1A, 1B, 1D, and 1E represent a relative percentage of zero-radius operational taxonomic units (ZOTUs). &nbsp;

**Table:** 

<b> 1A. Predicted Bacterial Phyla</b>	<b>Ruffles 3/18/25</b>	<b>Ruffles 3/26/25</b>	<b>Ruffles 4/20/25</b>	<b>Legolas 3/19/25</b>	<b>Legolas 3/25/25</b>	<b>Legolas 4/18/25</b>
Bacteroidetes	0.93	0.04	1.07	0.88	0.08	1.52
Pseudomonadota	6.23	24.45	24.33	14.18	0.14	32.94
Candidatus melainabacteria	0	0.01	0.01	0	0.02	0.02
Cyanobacteriota	0	0.01	0.02	0	0	0.06
Actinobacteria	0.01	0.16	0.02	0.02	0.13	0.04
Mycoplasmatota	0.31	0.02	0.08	0.23	0.09	0.21
Actinomycetota	0.88	6.28	1.11	1.05	2.13	1.49
Bacteroidota	1.31	7.49	3.33	1.77	6.85	4.72
Planctomycetes	0.03	0.02	0.06	0.05	0.09	0.05
Synergistetes	0.30	0.40	0.38	0.55	0.34	0.36
Synergistota	0.51	0.67	0.51	0.64	0.67	0.59
Chloroflexota	0.06	0.95	0.09	0.06	4.47	1.19
Bacillota	86.13	58.03	66.91	77.85	82.41	54.55
Planctomycetota	2.98	1.40	1.75	2.26	2.52	2.14
Thermodesulfobacteriota	0.21	0.07	0.32	0.31	0.07	0.11
Kiritimatiellota	0.11	0	0.01	0.14	0	0
<b>1B. Abundant Predicted Bacterial Genera</b>	Ruffles 3/18/25	Ruffles 3/26/25	Ruffles 4/20/25	Legolas 3/19/25	Legolas 3/25/25	Legolas 4/18/25
<i>Clostridium</i>	38.07	9.00	24.88	32.14	17.10	12.35
<i>Acinetobacter</i>	6.01	24.08	23.58	13.90	0	32.36
<i>Sarcina</i>	18.39	9.55	24.10	13.24	14.34	8.84
<b> 1C. Bacterial Information</b>	Ruffles 3/18/25	Ruffles 3/26/25	Ruffles 4/20/25	Legolas 3/19/25	Legolas 3/25/25	Legolas 4/18/25
Number of Sequences	28930	29883	29473	29234	29163	29930
Average Number of Sequences	.	29429	.	.	29442	.
Number of ZOTUs	403	394	409	417	398	416
Average Number of ZOTUs	.	402	.	.	410	.
<b>1D. Predicted Fungal Phyla</b>	Ruffles 3/18/25	Ruffles 3/26/25	Ruffles 4/20/25	Legolas 3/19/25	Legolas 3/25/25	Legolas 4/18/25
Basidiomycota	3.61	10.11	20.36	6.34	2.68	8.52
Ascomycota	96.39	89.89	79.64	93.66	97.32	91.48
<b>1E. Abundant Predicted Fungal Genera</b>	Ruffles 3/18/25	Ruffles 3/26/25	Ruffles 4/20/25	Legolas 3/19/25	Legolas 3/25/25	Legolas 4/18/25
<i>Candida</i>	1.55	75.16	21.71	2.50	92.28	67.81
<i>Phaeosphaeria</i>	8.08	2.57	6.77	13.72	0.71	5.11
<i>Parasarocladium</i>	54.04	1.14	1.45	5.96	0.93	1.26
<i>Paraconiothyrium</i>	5.78	1.74	7.34	10.54	0.68	3.72
<i>Cladosporium</i>	4.52	1.01	5.07	8.45	0.36	2.34
<i>Agaricus</i>	1.62	8.54	18.42	2.40	2.27	5.82
<i>Alfaria</i>	2.30	0.71	1.71	12.57	0.22	3.04
<i>Pestalotiopsis</i>	1.66	2.77	8.98	2.87	0.33	1.75
<b>1F. Fungal Information</b>	Ruffles 3/18/25	Ruffles 3/26/25	Ruffles 4/20/25	Legolas 3/19/25	Legolas 3/25/25	Legolas 4/18/25
Number of Sequences	32395	23967	27958	28265	33941	25394
Average Number of Sequences	.	28107	.	.	29200	.
Number of ZOTUs	197	171	186	206	156	186
Average Number of ZOTUs	.	185	.	.	183	.

## Description

Gopher tortoises ( *Gopherus polyphemus* ) are mostly found in the Southeastern United States and along the Coastal Plains. Gopher tortoises are listed as a threatened species by the State of Florida (Florida Fish and Wildlife Commission, 2025). &nbsp;These animals often burrow in dry sandy environments (Florida Fish and Wildlife Commission, 2025). &nbsp;However, they are subjected to numerous problems including habitat degradation and forced relocation. &nbsp;This can result in an unnatural mixing of the populations and an increased exposure to harmful parasites and disease (Huffman et al., 2018; Cozad&nbsp;et al., 2020; Folt&nbsp;et al., 2022; Whitfield&nbsp;et al., 2024; Jones&nbsp;et al., 2025). &nbsp;

&nbsp;

The diet of many tortoise species generally includes flowers, fruit, legumes, and several varieties of grass. (Abella and Berry, 2016; Figueroa et&nbsp;al., 2024). &nbsp;Though the diet of tortoises is well known, the information regarding the gut microbiome is lacking, which is not uncommon for reptilian herbivores (Sandri et&nbsp;al., 2020). &nbsp;This scarcity of information limits our understanding of the disease process and resilience of the animal.

&nbsp;

In this current study amplicon sequencing was performed to obtain an inventory of the bacterial and fungal diversity in the fecal material of two gopher tortoises, Ruffles and Legolas, living at the Peace River Wildlife Center in Punta Gorda, Florida. &nbsp;As a noninvasive technique, fecal samples are often utilized to examine the gut microbiome of animals (Tang et al., 2020).&nbsp; Three samples from each tortoise were collected over a one-month period of time. &nbsp;The average number of sequences and ZOTUs for Ruffles’ bacterial diversity was 29429 and 402, respectively. &nbsp;The average number of sequences and ZOTUs for Legolas’ bacterial diversity was 29442 and 410, respectively. &nbsp;The most abundant bacterial phylum in all samples was Bacillota (formerly Firmicutes), followed by Pseudomonadota (formerly Proteobacteria). &nbsp;The most predominant genus found in four of the samples was *Clostridium* (Table 1).

&nbsp;

In terms of fungi, the average number of sequences and ZOTUs for Ruffles was 28107 and 185, respectively. The average number of sequences and ZOTUs for Legolas was 29200 and 183, respectively. The most abundant fungal phylum throughout the samples was Ascomycota. *&nbsp;Candida* was the most abundant genera in four of the six samples (Table 1).

&nbsp;

Amplicon sequencing has been used to assess the fecal microbiome in different tortoise species. In the Seychelles giant tortoises ( *Aldabrachelys gigantea* )&nbsp;living in different environments the most abundant phyla were Bacteroidota (42.00%), Bacillota (32.00%), and Spirochaetota (9.00%) (Sandri et&nbsp;al., 2020). In desert tortoises Bacillota, Bacteroidota, and Actinobacteroidota comprised the majority of the phyla found in the samples (Blair et al., 2025). In captive Bolson tortoises ( *Gopherus flavomarginatus* ) the most abundant phylum was Bacillota followed by Fibrobacterota (García-De La Peña et al., 2019). &nbsp;In a previous study examining the bacterial diversity in the fecal material of a south-central Florida population of gopher tortoises, the most predominant phyla were the Bacillota (36.00%) and Bacteroidetes (36.50%) (Yang et al., 2015).&nbsp; In a more recent study examining a population living in Abacoa Greenway in Jupiter, Florida, 20 different phyla were identified with Bacillota (70.44%) as the most predominant (Giakoumas et al., 2024).&nbsp; In our study, the average percentage of Bacillota for Ruffles was 70.35, and for Legalos was 71.60 which is much higher than detected in the study completed by Yang et al., (2015), but very similar to the results found in the study Giakoumas et al., (2024). &nbsp;Our findings were more consistent with those of previous studies conducted on herbivorous reptiles.&nbsp; For example, in the Galapagos tortoise ( *Chelonoidis nigra* ), the green iguana ( *Iguana iguana* ), and the marine iguana ( *Amblyrhynchus cristatus* ) the relative abundance of Bacillota was 81.10%, 74.00% and 75.10%, respectively (Hong et al., 2011).

&nbsp;

In our study, in terms of fungal diversity there were only two phyla present, Ascomycota and Basidiomycota (Table 1).&nbsp; In previous gopher tortoise studies, the fungal diversity was not examined.&nbsp; It has been documented that in the fecal material of reptiles these two phyla are seldomly found (Weber, 1970).&nbsp; Ascomycota and Basidiomycota are major decomposers of plant cell walls. &nbsp;Specifically, Ascomycetes are primarily responsible for the digestion of cellulose and hemicellulose, whereas Basidiomycetes are more active in lignin degradation (Manici et al., 2024). &nbsp;These fungi are commonly detected in the gastrointestinal tracts of animals and often dominate the gut mycobiota, particularly in herbivores consuming a plant-rich diet (Zhao et al., 2023; Lv et al., 2023).

&nbsp;

The breakdown of dietary cellulose and hemicellulose can also be accomplished by cellulolytic bacteria.&nbsp; Many such bacteria that are found within the phyla Bacillota as well as Bacteroidetes are capable of cellulose digestion (Bhatia et al., 2024). The gopher tortoise is a hindgut fermenter that utilizes microbial fermentation of starch in the hindgut.&nbsp; Unnamed bacterial taxa likely play an important role in the metabolism of the animal and warrant further investigation (Giakoumas et al., 2024).&nbsp; However, this is also likely true of the fungal taxa.

&nbsp;

Finally, it is well known animals can be reservoirs for pathogens.&nbsp; In a previous study involving gopher tortoises the potential pathogens Mycoplasmaceae sp., and *Helicobacter* sp. were detected (Giakoumas et al., 2024).&nbsp; These bacterial species were not detected in this current study.&nbsp; In our study we detected *Candida tropicalis* with a range of 1.41–75.00% of the sequences for Ruffles and 2.31-92.20% of the sequences for Legolas.&nbsp; This zoonotic fungal species has been detected in the Puff adder ( *Bitis arietans* ), the Moroccan cobra ( *Naja haje legionis* ) (Ugochukwu et al., 2024), bats (Pinto et al., 2025), cetaceans (Garcia-Bustos et al., 2024), cows (Clarke, 1960), goats, sheep, psittacines, rhea monkeys, horses, sirenians, and shrimp (Cordeiro Rde et al., 2014).&nbsp; This yeast can cause mastitis in cows (Clarke, 1960), blood stream infections in humans (Guinea, 2014) and oropharyngeal candidiasis (Collins et al., 2011).&nbsp; In conclusion, our study reaffirms the role of animals as reservoirs for zoonotic microorganisms.&nbsp; These findings underscore the importance of continued surveillance of microbial communities in animals to better understand pathogen distribution and potential risks to human health.

## Methods

Animals and sample collection&nbsp;

The two gopher tortoises in this study live at the Peace River Wildlife Center Punta Gorda, Florida.&nbsp; Ruffles is a 61-year-old female, with severe metabolic bone disease (MBD) from many years of incorrect husbandry which has resulted in a disfigured shell.&nbsp; The shell length is 9 inches. &nbsp;Legolas is a female of unknown age with a shell length of 14.5 inches.&nbsp; Three samples from each tortoise were collected over an approximate one-month period of time and placed into separate containers.&nbsp; The date of sampling is found in Table 1.&nbsp; All samples were transported and then stored in a − 40 °C freezer until used.

&nbsp;

Microbiota analysis

The bacterial diversity and the fungal diversity within the six-gopher tortoise fecal samples used for our study were determined using bTEFAP® services. &nbsp;These assays were performed by MR DNA (Shallowater, TX). &nbsp;Primers 515F-806 (forward: GAG TTT GAT CNT GGC TCA G;

reverse: GTN TTA CNG CGG CKG CTG) and ITS1-2 (forward: CTT GGT CAT TTA GAG GAA GTA A; reverse: GCT GCG TTC TTC ATC GAT GC) were used to amplify bacterial and fungal DNA, respectively. A more detailed explanation of the methods can be found in the study by Sanchez et al., 2021.
